# Glucose-Lowering Agents Developed in the Last Two Decades and Their Perioperative Implications

**DOI:** 10.3390/ph18010004

**Published:** 2024-12-24

**Authors:** Basavana Goudra, Geno J. Merli, Michael Green

**Affiliations:** 1 Jefferson Surgical Center Endoscopy, Department of Anesthesiology, Sidney Kimmel Medical College, Thomas Jefferson University, Philadelphia, PA 19107, USA; 2 Jefferson Vascular Center, Division of Vascular Medicine, Sidney Kimmel Medical College, Thomas Jefferson University, Philadelphia, PA 19107, USA; geno.merli@jefferson.edu; 3 Department of Anesthesiology and Perioperative Medicine, Sidney Kimmel Medical College, Thomas Jefferson University, Philadelphia, PA 19107, USA; michael.green2@jefferson.edu; 4 Enterprise Perioperative Services, Jefferson Health System, Philadelphia, PA 19107, USA

**Keywords:** GLP-1RA, SGLT-2i, gliptins, Icodec, IcoSema, Imeglimin, dipeptidyl peptidase-4 inhibitors, DPP-4i

## Abstract

The last two decades have provided far more options f both patients and their physicians in the treatment of diabetes mellitus. While dipeptidyl peptidase-4 inhibitors (DPP-4is) and glucagon-like peptide 1 receptor agonists (GLP-1RAs) have been approved for nearly two decades, sodium–glucose cotransporter 2 inhibitors (SGLT-2is) are relatively new. Of interest to perioperative physicians, these drugs present specific perioperative concerns, prompting many societies to issue guidelines. Retained gastric contents due to slow gastric emptying is a significant drawback of GLP-1RAs, increasing the risk of aspiration. Recommendations include withholding GLP-1RAs for a predefined period of time, performing gastric ultrasound to evaluate gastric contents, modifying anesthesia management, particularly with regard to the airway, or canceling the scheduled (elective) surgery or procedure. SGLT-2is are known to increase the risk of euglycemic ketoacidosis. The benefits of both GLP-1RAs and SGLT-2is extend beyond the treatment of diabetes. As a result, perioperative physicians may encounter their use outside of their traditional indications. SGLT-2is are being used extensively to treat heart failure and obesity, for example. There have been other developments as well. For instance, Imeglimin, a variant of metformin available in Japan and India, Icodec, a once-weekly basal insulin formulation, and IcoSema, a once-weekly combination of Icodec plus semaglutide, are all being explored, although in their early stages or facing approval challenges.

## 1. Introduction

With an increase in the worldwide prevalence of diabetes, better tools have become available to manage the condition. Many new classes of drugs have become available that offer remarkable flexibility for managing both type 1 and type 2 diabetes mellitus (T1DM and T2DM). The first of this new class of T2DM drugs, approved by the FDA in 2005, is glucagon-like peptide-1 receptor analogs (GLP1-As), for example, exenatide. DPP-4 inhibitors (also known as gliptins) were approved in 2006. The first sodium–glucose cotransporter-2 inhibitors (SGLT-2is) were approved in 2013. Some of these drugs have become extremely popular for weight reduction alone in the absence of diabetes mellitus. SGLT-2is are used in the treatment of heart failure.

In addition, once-weekly basal insulin analog insulin (Icodec) is becoming popular in some countries (although not available in the USA), and the once-weekly combination of insulin Icodec plus semaglutide (a GLP-1A) is likely to be available in the very near future.

A sufficient body of evidence has been gathered to better understand the perioperative implications of some of these drugs. While pulmonary aspiration is a major potential risk with GLP-1As, SGLT2is predispose patients to euglycemic ketoacidosis. This current review will explore the existing research with regard to these drugs and their interactions, as it relates to anesthesia and perioperative management.

## 2. Glucagon-like Peptide 1 Receptor Agonists

### 2.1. Physiology, Pharmacology, and Mechanism of Action

Glucagon is a polypeptide consisting of 29 amino acids in a single chain secreted by the alpha cells of pancreatic islets. Glucagon stimulates glycogenolysis and gluconeogenesis, and with these two actions, plays an important role in glucose homeostasis [1]. Glucagon-like peptide 1 (GLP1), on the other hand, is a 30-amino acid peptide hormone produced in the lower intestinal epithelial endocrine L-cells that resemble pancreatic alpha cells by the differential processing of proglucagon [2,3]. Along with glucose-dependent insulinotropic polypeptide, these two polypeptides are classified as incretins. Incretins are hormones that are secreted in response to the presence of food in the gastrointestinal (GI) tract.

Proglucagon is the precursor of both glucagon and GLP1. In smaller amounts, GLP1 is also secreted in the pancreas and central nervous system. As stated, it is primarily secreted in response to a meal [4] (along with gastric inhibitory polypeptides from the K-cells of the upper small intestine), and the secretion corelates with insulin secretion [5]. An enzyme named dipeptidyl peptidase 4 (DPP-4) located on the luminal surface of the endothelial cells destroys three-fourths of GLP1 before it reaches portal circulation [6,7]. Nearly half of the remaining GLP1 is destroyed in the liver, so that eventually only about 10–15% of the secreted GLP1 actually enters systemic circulation.

Among the multitude of actions of GLP1 are the glucose-dependent stimulation of insulin secretion, the slowing of gastric emptying, the inhibition of food intake, increased natriuresis, a reduction in blood pressure, neuroprotection, and diuresis [8]. Recently, it has also been implicated in learning and memory, reward behavior, and palatability. It has cardio- and neuroprotective effects, and decreases inflammation and apoptosis. As a result, GLP1 agonists have an expanding role in clinical practice beyond the treatment of T2DM. These include Alzheimer’s dementia, hypertension, dyslipidemia, non-alcoholic steatohepatitis, parkinsonism, infertility, polycystic ovarian syndrome, associative learning, stroke, and even some types of cancer. They are increasingly used to treat New York Heart Association class III/IV heart failure [9]. In these patients, they are shown to improve left ventricular function, functional status, and quality of life. In addition, major cardiovascular events seem to be lower in patients treated with GLP1 analogs. As a result, an anesthesia provider should expect to see patients being prescribed these drugs for a wide variety of indications.

### 2.2. Perioperative and Anesthesia Implications

Since the approval of the first glucagon-like peptide-1 receptor analog (GLP1A), exenatide, by the FDA in 2005, the market for GLP1As has exploded. Some of the popular GLP1As available on the market are Trulicity, Mounjaro, Wegovy, Zepbound, Saxenda, and Victoza. According to Prophecy Market insights, the global GLP1A market size and share is projected to grow from USD 45.3 Billion in 2023 to reach USD 606.3 Billion by 2034 [10].

### 2.3. Gastric Emptying and GLP1A

Of particular significance to the anesthesia providers is the ability of GLP1As to slow stomach emptying. They are known to inhibit prandial gastrointestinal motility through myenteric neuronal mechanisms [11]. They also decrease gastric acid secretion, GI transit, motility, and gastric wall tone [12,13,14,15,16,17]. The gastric slowing effect is likely to be a vagal effect, as the effect is lost following vagal afferent denervation [18].

Many studies have quantified the delayed gastric emptying effects of GLP1A [19,20,21]. These methods have included the use of radioactive tracers, acetaminophen-based absorption testing, stable isotope breath testing, esophagogastroduodenoscopy (EGD), and capsule studies.

In a placebo controlled study, Stevens et al. assessed gastric emptying by scintigraphy in 15 volunteers on two occasions following 2 days of dosing with sitagliptin (100 mg/day) or placebo [19]. They did not find any difference in gastric emptying. In another study, Maselli et al. conducted a randomized, parallel-group, placebo-controlled, 16-week trial of liraglutide. Among other parameters, they measured the gastric emptying of solids and gastric volumes [20]. In their analysis, liraglutide prolonged both 50% and 25% gastric emptying compared with the placebo (at 5 and 16 weeks) and increased satiety at 16 weeks. After 8 weeks of lixisenatide treatment, Rayner et al. observed sustained slowing of gastric emptying in T2DM patients [21].

However, more relevant for anesthesia providers would be studies involving retained gastric contents visualized during EGD. In a single-center retrospective electronic chart review of 404 patients (of which 33 were on semaglutide) undergoing elective esophagogastroduodenoscopy, Silveira et al. found increased residual gastric content (RGC) in 8 patients (24.2%) in the semaglutide group and 19 (5.1%) in the non-semaglutide group [22]. In total, 12.2% of these patients were taking semaglutide for weight loss, and the rest for T2DM. All the patients were appropriately fasting. In those with increased RGC, semaglutide was withheld for 10 (6–15) days. In those without increased RGC, it was withheld for 11 (7.75–12.5) days (*p* = 0.54). RGC was defined as any amount of solid content from the esophagus to the pylorus, or >0.8 mL/Kg of fluid content as measured from the aspiration/suction canister. One patient in the semaglutide group sustained pulmonary aspiration; however, the person had previous gastric bypass.

After investigating the patients taking GLP1 analogs (liraglutide, dulaglutide, semaglutide, and semaglutide) for diabetes mellitus, and undergoing EGD, Kobori at al concluded that GLP1 analogs were associated with increased gastric residue [23]. In this case–control study, which involved propensity score-matched comparison, the GLP-1A group exhibited higher gastric residue, which was statistically significant.

However, in another retrospective cohort study with matched controls, Stark et al. did not find that GLP1A significantly increased the odds of retained food on EGD [24].

HIramoto et al. conducted a meta-analysis that included fifteen studies [25]. Of these, five studies utilized gastric emptying scintigraphy, while ten studies utilized the acetaminophen absorption test. They did not find any substantial differences in gastric emptying on the modalities reflective of liquid emptying. Despite the fact that the administration of GLP1A was associated with higher gastric emptying half-time on scintigraphy (T_1/2_) than placebo, its clinical impact was only limited. Considering that most patients presenting for elective procedures fast for 8–10 h, the clinical impact is minimal or there is none.

### 2.4. Society Guidelines

Various anesthesia and gastroenterology societies have put forth their guidelines to assist clinicians in their practice [26,27,28,29,30,31].

The American Society of Anesthesiologists (ASA) task force on preoperative fasting published their guidelines in June 2023. For elective procedures (surgical and non-surgical), the organization recommends withholding GLP-1 agonists on the day of the procedure/surgery (if the patient is taking daily doses). For those on once-weekly dosing, the recommendation is to withhold it a week prior to the procedure/surgery. This applies to patients taking their GLP-1 agonists for any indication. The guidelines from other anesthesia societies are similar. In a detailed, evidence-supported communication to its members, the American Gastroenterological Association (AGA) advised exercising best practices when performing endoscopy on patients on GLP1As. The AGA argues that the ASA’s “consensus-based guidance on perioperative management” is not based on sufficient and robust evidence. The AGA also argues that there are “No data to support stopping GLP-1 agonists prior to elective endoscopy”.

Finding liquid/semisolid/solid contents in the stomach after esophageal intubation is not uncommon during EGD. Lin et al. studied the practice patterns and outcomes of patients with retained gastric food content encountered during endoscopy [32]. In total, 4.1% (730/17,868) of the patients who underwent endoscopy at their facility (Loma Linda University Medical Center, Loma Linda, CA, USA) had retained gastric food contents. All the patients were fasting for at least 6 h for solids and 2 h for clear liquids. Exclusion of those with altered surgical anatomy or who were already mechanically ventilated before the procedure left 629 (3.5%) patients. Of these, 506 received moderate sedation, while the remainder were sedated by an anesthesia provider administering general anesthesia. Altogether, 40 (6.4%) had respiratory adverse events, including aspiration pneumonia. One patient had a respiratory obstruction requiring mucosal plugging. Sadly, 21 (51.2%) required admission to the intensive care unit and mechanical ventilation, with 10 of them dying. As demonstrated here and in other studies, it is not uncommon to encounter retained gastric food in the stomach of patients presenting for EGD (in the absence of any GLP1 A administration), which can lead to aspiration pneumonia [33,34,35,36,37]. There are established risk factors, such as gastroparesis, and anesthesia providers do not intubate or delay such patients on a routine basis.

In conclusion, the guidelines issued by anesthesia societies seem to allow for a cautious approach to an evolving problem. The American diabetes association concedes that there are little data in the area of delayed gastric emptying in the perioperative period [38]. While waiting for prospective studies in patients presenting for an endoscopy, both in relation to the length the patients were on the GLP1As and the duration of withholding, we agree that patients arriving for GI endoscopy should be treated differently than those scheduled to undergo a major surgical procedure. Those presenting for a combined EGD and colonoscopy should have their EGD performed first. This provides an opportunity to carefully pass the endoscope into the stomach in a semi-sitting position. Any fluid visualized can be suctioned, and the procedure/s can proceed. If solids/semisolids are seen, the planned procedures should be aborted Although most hospitals have accepted and adapted the new advisory in terms for withholding these medications, patients can occasionally present to a procedure either unaware of the recommendations or having forgotten to follow them. In such cases, it is important to explain the current state of knowledge/recommendations and adopt a prudent approach that best serves the needs of our patients. Extended fasting times in patients who present for colonoscopy or combined EGD/colonoscopy should provide an additional layer of protection.

The most recent multi-society guidelines (that included ASA), released on 29th October 2024, recommend a shared decision-making approach in consultation with procedural, anesthesia, and prescribing care teams balancing the need for GLP-1As with individual patient risk [39]. The guidelines noted that the escalation phase of GLP-1A causes a greater delay in gastric emptying. Additionally, higher doses and weekly dosing regimens are more likely to cause gastrointestinal side effects. In the absence of any inherent factors that slow gastric emptying, one might not withhold GLP-1A. The factors noted above, such as preoperative liquid diet for at least 24 h (as recommended in patients undergoing colonoscopy and bariatric surgery), may be extended to other patients with intrinsic risk factors such as gastroparesis. Point-of-care gastric ultrasound to evaluate gastric contents should be utilized as appropriate.

## 3. Sodium–Glucose Cotransporter-2 Inhibitors

### 3.1. Physiology, Pharmacology and Mechanism of Action

Sodium–glucose cotransporter-2 (SGLT-2) inhibitors, also called gliflozins, such as canagliflozin, dapagliflozin, and empagliflozin, are popular in the treatment of both type 1 and T2DM [40,41,42]. In addition, their role in the management of obesity and heart failure has been acknowledged [43,44].

The most recent physiological and pharmacological aspects of SGLT-2 inhibitors are extensively discussed by Ernest M Wright from the Department of Physiology, David Geffen School of Medicine at UCLA, Los Angeles, California [45]. Typically, of 120–180 g of glucose filtered in the kidney every 24 h, only 0.5 g is excreted in the urine. SGLT2 receptors are principally found in the proximal convoluted tubule of the nephron [44]. Nearly 95% of the filtered glucose is reabsorbed by these nephrons. The SGLT2 receptors are upregulated in patients with diabetes mellitus, further increasing the absorption. The remaining glucose is reabsorbed by the SGLT1 activity present in the S3 segment pf the nephron, which consists of the remainder of the proximal straight tubule in the medullary ray and the outer stripe of the outer medulla [46].

By potent and selective SGLT-2 receptor inhibition, the gliflozins induce dose-dependent glucosuria in healthy subjects [47]. However, even when working at their full therapeutic doses, they only decrease glucose reabsorption by 40–50%. As a result, even though a large portion of filtered glucose is normally reabsorbed by SGLT 2 receptor mediated action, the resulting glucosuria is modest, about 50–60 g in 24 h [48]. Efforts are being made to create SGLT1 inhibitors that can further increase glucosuria. Dual SGLT1/SGLT2 inhibitors such as sotagliflozin are available [49,50,51,52]. It is approved for both heart failure and diabetes mellitus. However, the maximum glucose excretion reported with this drug is 44 g. This is less than that reported with some SGLT2 inhibitors, which can be as high as 60–70 g per day.

In addition to producing glucosuria, the inhibition of SGLT-2 receptors is known to increase the plasma glucagon/insulin ratio in mice in the fasted state [53]. SGLT-2 receptors are not expressed in pancreatic α- and β-cells in mice. However, they are expressed in human pancreatic α-cells. The cause of altered glucagon/insulin ratio is indirect and not related to the direct effect on glucagon and insulin secretion.

SGLT-2 inhibitors are used frequently in conjunction with other drugs, such as metformin and glucagon-like peptide 1 receptor agonists (GLP1-As) in T2DM. In addition, newer indications are being added on a regular basis.

The use of SGLT-2 inhibitors in type 1 diabetes mellitus (T1DM) is relatively recent and is controversial [41]. The risk of ketoacidosis increases with combined insulin-SGLT-2 inhibitor therapy [40]. However, a meta-analysis of 16 RCTs consisting of 7192 patients with T1DM found a reduction in the insulin requirements without an increased risk of hypoglycemia and diabetic ketoacidosis [41]. Nevertheless, in another meta-analysis of seven randomized trials encompassing 42,375 participants and 5 cohort studies, encompassing 318,636 participants with T2DM, SGLT2 inhibitors were found to increase the risk of ketoacidosis. Clearly, the risk of ketoacidosis should be explained to these patients.

SGLT2 inhibitors are effective in facilitating weight loss on their own, and combining them with other agents provides additional benefits in some patients. Pratama et al. performed a systematic review of seven studies and concluded that SGLT2 inhibitors can cause significant weight loss in patients with obesity and without diabetes mellitus [54]. Bays et al. evaluated the effect of canagliflozin, an SGLT-2 inhibitor in overweight and obese subjects over 12 weeks, at different doses (overweight and obese subjects) administered once daily. One of the side effects observed was higher rates of genital mycotic infections in women. Patients also experience significant weight loss at the end of the first 2 weeks [55]. Patients who are >70 years old, with a body mass index >25 kg/m^2^, and those using sulfonylureas have better odds of losing weight [56]. SGLT-2 inhibitors have demonstrated the ability to reduce body weight and visceral adiposity in Asian patients with T2DM [57].

In adults with type 1 diabetes mellitus, Sotagliflozin, an SGLT-2 inhibitor, when used as an adjunct to insulin therapy, significantly reduced the predicted 5- and 10-year cardiovascular disease risk, possibly by reducing body weight and blood pressure [58].

There is evidence to suggest that SGLT-2 inhibitors can prevent heart failure (HF) in patients with T2DM and decrease major adverse cardiovascular events and hospitalization for HF in patients with concomitant HF and T2DM [59,60]. Dapagliflozin, an SGLT-2 inhibitor, was shown to reduce the combined risk of worsening heart failure or cardiovascular death among patients with heart failure and mildly reduced or preserved ejection fraction [61]. This study included 6263 patients with heart failure and a left ventricular ejection fraction of more than 40%. A meta-analysis of 12,251 participants supported the benefits of SGLT-2 inhibitors in reducing the risk of cardiovascular death and hospitalizations in patients with heart failure irrespective of ejection fraction or care setting [62]. Further, the beneficial effects of these drugs in heart failure seems to be a class effect [44]. Some of the mechanisms could be indirectly related to diuretic-like effects, beneficial effects on calcium handling, and decreases blood pressure and body weight; however, there could be direct molecular mechanisms at play, and these are being explored.

### 3.2. Perioperative and Anesthesia Implications

The inhibition of SGLT-2 receptors is known to increase the plasma glucagon/insulin ratio in fasting mice. The inhibition of the glucose transporter SGLT2 with dapagliflozin in pancreatic alpha cells is also known to trigger glucagon secretion [63]. In patients with T1DM, increased glucagon secretion contributes to the elevated ketones and acidosis present in diabetic ketoacidosis [64].

Patients presenting for elective surgical procedures routinely fast for varying periods of time, as per the recommendations of the ASA [65]. In addition, patients presenting for colonoscopy fast for extended periods of time for semisolids and solids. Although recent changes have made the colonic preparation more tolerable, the duration of abstaining from solids has stayed the same [66].

Cases of euglycemic ketoacidosis in the perioperative period are reported across all specialties and in various perioperative settings [67,68,69,70,71]. Mackintosh et al. reported a case of euglycemic ketoacidosis (EDKA) that presented as unexplained encephalopathy in neurocritical care after the resection of a left temporal meningioma [68]. The 68-year-old woman with a history of T2DM diabetes mellitus had not taken empagliflozin the day of surgery; however, the drug has a half-life of >12 h. Biochemistry revealed blood glucose levels of 140 to 160 mmol/L, bicarbonate of 9 mmol/L, an anion gap of 21, and a pH of 7.2. Treatment with insulin was effective and restored the patient’s neurological status to baseline.

Ritche et al. reported a case of intraoperative severe sodium–glucose cotransporter-2 (SGLT-2) inhibitor-associated EDKA requiring treatment in the critical care unit in a patient undergoing surgery for a fracture of the intracapsular neck of the femur. The patient was on Empagliflozin 10 mg, once a day. In the operating room, a severe, high anion gap metabolic acidosis (base excess of −22, pH of 6.96, and ketones 4.9 mmol/L, and a normal lactate level) was noted. The patient was successfully managed with intravenous insulin and 0.9% sodium chloride [67] with inescapable morbidity due to delayed mobilization.

Kameda et al. reported ketoacidosis without elevated blood glucose in a 65-year-old woman with type II diabetes mellitus and unstable angina who underwent a semi-emergency coronary artery bypass grafting. The SGLT2 inhibitor was stopped for more than 24 h preoperatively [69].

Meyer et al. reported eight cases (aged between 45 and 75 years) of SGLT2i-associated EDKA in the setting of colonoscopy that occurred between August 2019 and February 2020 across three centers [72]. Seven patients took SGLT2i up to the day prior and one took it on the morning of colonoscopy. Factors such as very-low-calorie diet, volume depletion (related to fasting and preparation), and reduced insulin administration/secretion are some of the purported factors.

Blau et al. analyzed the data from an FDA adverse event reporting system for reports of acidosis in patients treated with canagliflozin, dapagliflozin, or empagliflozin [73]. They discovered 192 reports of ketoacidosis (of a total of 259 reports of acidosis) in patients on SGLT2 inhibitors. Chumbe et al. observed that the use of SGLT-2is at least a week prior to colonoscopy significantly increased the risk of ketoacidosis post procedure [71]. The FDA revised their drug label with warnings in September 2023 to include prolonged diabetic ketoacidosis and glucosuria [74,75].

### 3.3. Society Guidelines

In line with the FDA recommendations, the American college of cardiology recommends that canagliflozin, dapagliflozin, and empagliflozin should be discontinued 3 days before scheduled surgery, and that ertugliflozin should be stopped at least 4 days prior to surgery [76]. In the perioperative setting, EDKA should be considered when a patient has an anion gap metabolic acidosis and pH < 7.3, elevated ketones in the blood or urine, and blood glucose < 200.

The Australian diabetic society recommend that clinicians should consider DKA/EDKA in patients taking SGLT2is and exhibiting symptoms such as abdominal pain, nausea, vomiting, fatigue, or metabolic acidosis with relatively normal plasma glucose or capillary blood ketone (or blood beta-hydroxybutyrate) levels > 1.0 mmol/L with or without hyperglycemia and base excess (BE) < −5 mmol/L [77]. Further, they recommend withholding SGLT2is at least 3 days pre-procedure for surgeries and procedures requiring one or more days stay in hospital, and/or requiring ‘bowel preparation’, including colonoscopy.

The American diabetes association (ADA) agrees that SGLT2 inhibitors should be avoided in cases of severe illness, in people with ketonemia or ketonuria, and during prolonged fasting and surgical procedures. In patients with heart failure, they may be initiated or continued during hospitalization and upon discharge, if there are no contraindications, and after recovery from the acute illness. Further, regarding their use during the perioperative period, the ADA advises considering the discontinuation of SGLT2 inhibitors 3–4 days before surgery [38].

At the time of writing, with reference to SGLT2 inhibitors, the ASA has not issued an official statement.

### 3.4. Postoperative Concerns

Diabetic Ketoacidosis: SGLT-2 inhibitors are associated with an almost three-fold increased risk of diabetic ketoacidosis. Assess patients with clinical features for ketoacidosis. If suspected, discontinue the SGLT2 inhibitor, and evaluate and treat the patient promptly. Before initiating an SGLT2 inhibitor, consider the risk factors for ketoacidosis. Clinicians should consider temporarily discontinuing SGLT2 inhibitors in clinical situations that may predispose the patient to DKA. The risk for diabetic ketoacidosis is highest for canagliflozin, followed by empagliflozin and dapagliflozin.

Euglycemic DKA: Euglycemic DKA is a triad of increased anion gap acidosis, the presence of ketosis, and serum glucose < 250 mg/dL. SGLT-2 inhibitors appear to be associated with euglycemic DKA, possibly due to their noninsulin-dependent glucose clearance, hyperglucagonemia, and volume depletion. Ertugliflozin and canagliflozin have been associated with euglycemic DKA. However, other SGLT-2 inhibitors also carry risk for euglycemic DKA.

Acute Kidney Injury: AKI may occur with the initiation of SGLT2 therapy due to intravascular volume contraction. Before initiating an SGLT2 inhibitor, evaluate and correct the volume status, especially in elderly patients receiving diuretics and those with renal impairment. A recent pharmacovigilance study conducted using the FDA’s adverse event reporting system (FAERS) database revealed that canagliflozin had a significant association among SGLT2 inhibitors with acute kidney injury. Patients aged >65 were also identified as being at high risk for developing AKI.

Hypoglycemia: The risk of hypoglycemia is increased with the SGLT2 concomitant administration of insulin secretagogues, such as sulfonylureas or insulin. Physicians should reduce the dose of the insulin secretagogue or insulin when combined with SGLT2 inhibitors. Studies indicate that the risk of hypoglycemia is higher in elderly patients taking SGLT2 inhibitors. Use it with caution.

Fournier Gangrene: Fournier gangrene is a rare but life-threatening necrotizing fasciitis of the perineum demanding urgent surgical intervention. Cases have been reported in males and females. Its clinical features include fever, pain, tenderness, and swelling in the genital or perineal region. Serious outcomes of it include hospitalization, surgeries, and death. Risk factors include pre-existing diabetes, alcohol use, hypertension, advanced age, obesity, smoking, liver failure, immunocompromised conditions (HIV, inflammatory bowel disease, malignancy), end-stage renal disease, and radiotherapy. Iatrogenic procedures in the genital area, piercings, implants, and IV drugs use can be portals of entry for microorganisms.

Most FG infections are polymicrobial; therefore, an antibiotic cocktail should cover streptococcus, MRSA, pseudomonas, coliforms, Bacteroides, and Clostridium. Fungal superinfection requires appropriate coverage with amphotericin B and other antifungal agents. Emergent surgical debridement, broad-spectrum antibiotics, and resuscitation with IV fluids and vasopressors are the cornerstones of treatment.

## 4. Dipeptidyl Peptidase 4 Inhibitors

### 4.1. Physiology, Pharmacology, and Mechanism of Action

Dipeptidyl peptidase IV (DPP-4) inhibitors or gliptins were second among the new generation of antidiabetic drugs to be approved by the FDA. Sitagliptin Phosphate (Januvia, Merck) was approved in October 2006 [78].

The enzyme DPP-4 is present in many tissues, and it is a membrane-bound serine proteinase (a type of protease) that removes dipeptides from the amino terminal end of peptides [79]. In addition to incretin hormones such as GLP-1 and GLP-2, DPP-4 also cleaves growth factors, chemokines, and peptides [80]. Incretins (e.g., GLP-1) stimulate insulin secretion and are secreted in response to food intake. As a result of this “incretin effect”, oral intake of glucose produces a stronger (two- to three-fold higher) insulin secretory response compared to intravenous glucose administration [81]. The inhibition of DPP IV activity followed by reduced peptide cleavage and increased endogenous incretin hormone activity is the main rationale for the use of gliptins in the treatment of T2DM [82]. It is important to note that DPP-4 inhibitors have no intrinsic ability to lower glucose levels and carry no risk of hypoglycemia.

The HbA1c lowering ability of DPP-4 inhibitors in patients with T2DM is studied extensively. In a meta-analysis of 43 randomized controlled trials, Esposito et al. found that a greater proportion of T2DM patients can achieve the HbA1c goal of <7% with DPP-4 inhibitors compared to a placebo. In addition, there was no weight gain or hypoglycemic risk [83]. In another meta-analysis of eight RCTs of DPP-4 inhibitors and metformin (as an initial combination therapy or monotherapy) in patients with T2DM, DPP-4 inhibitors were found to be safe and effective in controlling blood glucose [84].

On the positive side, DPP-4 inhibitors do not cause gastrointestinal side effects such as nausea [85]. They do not have cardiovascular benefits and in fact might cause congestive heart failure by the degradation of B-type natriuretic peptides. In comparison to SGLT2i and GLP1 analogs, DPP-4 inhibitors only produce modest reductions in A1c levels (0.5–0.8). Although DPP-4 inhibitors lack significant cardiovascular benefit in T2DM, for patients with T2DM, other than atrial flutter, they do not cause any major cardiac arrhythmias. In short, although their efficacy is less than GLP-1 receptor agonists and SGLT2i, and they lack concomitant extra diabetic benefits, they are relatively safe [86]. Nasopharyngitis and skin lesions are some of the reported adverse effects; however, these are not serious enough to discontinue the treatment [85]. Neither DPP-4 inhibitors nor GLP-1RAs are known to pose any demonstrable risk of pancreatitis or pancreatic cancer [87].

### 4.2. Perioperative and Anesthesia Implications

The working party of the Association of Anaesthetists of Great Britain and Ireland allows DPP-4 inhibitors to be taken by the patient as normal on both the day preceding surgery and the day of surgery. Those patients on variable-rate intravenous insulin infusion should stop DPP-4 inhibitors until they are eating and drinking normally [88]. The ADA does not endorse the administration of DPP-4 inhibitors in hospital to patients [38].

## 5. Imeglimin

### 5.1. Physiology, Pharmacology, and Mechanism of Action

Imeglimin is the most recent among the new classes of drugs employed in the treatment of diabetes mellitus. Although as of yet not approved by the FDA, Imeglimin was approved in Japan in June 2021, and India in September 2022 [89].

Imeglimin is structurally similar to metformin [90]. It is synthesized from metformin by adding acetaldehyde to a solution of metformin hydrochloride, sodium hydroxide, and water [91]. It is known to reduce blood glucose by two mechanisms. It amplifies glucose-stimulated insulin secretion and preserves β-cell mass. It also enhances insulin action, inhibits liver glucose output, and improves insulin signaling in both liver and skeletal muscle. At the cellular level, it acts by modulating the mitochondrial function. More specifically, it competitively inhibits complex I of the respiratory chain while restoring the function of complex III [91].

Imeglimin is seen as a valuable addition to the T2DM treatment arsenal. In a Japanese phase 3, pivotal, open-label trial, patients with T2DM were prescribed with Imeglimin 1000 mg twice daily, orally, for 52 weeks as monotherapy or combination therapy (with one of the following: -α-glucosidase inhibitor, biguanide, dipeptidyl peptidase-4 inhibitor, glinide, glucagon-like peptide-1 receptor agonist, sodium–glucose co-transporter-2 inhibitor, sulphonylurea, or thiazolidinedione. The reduction in HbA1c was modest both as mono and oral combination therapy (0.46% and 0.56–0.92%). The most effective HbA1c reduction occurred with DPP4 inhibitor in combination with Imeglimin [92]. More importantly, no clinically significant changes in ECG, vital signs, physical examination, or laboratory tests were noted in any groups. The reduction in HbA1c improved significantly in poorly controlled T2DM when Imeglimin was added to their insulin regime.

In a meta-analysis of eight RCTs, Abdelhaleem et al. found that the Imeglimin group was superior to the control group in controlling glycated hemoglobin and fasting plasma glucose (*p* < 0.00001). However, no benefits were noted with regard to lipid parameters, including triglyceride, LDL-C, HDL-C, and a homeostasis model assessment of insulin resistance (HOMA-IR), a method for estimating insulin resistance [93]. In another meta-analysis of three double-blind RCTs, Singh et al. found a significant reduction in HbA1c with Imeglimin 1000 mg BID (compared to a placebo); however, this showed high heterogeneity [89].

Imeglimin was safely used in six patients with T2DM undergoing hemodialysis or peritoneal dialysis. There were no adverse effects, such as hypoglycemia, diarrhea, nausea, or vomiting [94]. Imeglimin has shown favorable effects on the development of plaque formation and the progression of atherosclerosis in mice, in whom atherosclerotic plaque formation was induced artificially by producing hyperglycemia with streptozotocin treatment [95]. In mice studies, Imeglimin was also shown to prevent heart failure while preserving ejection fraction [96].

### 5.2. Perioperative and Anesthesia Implications

Currently, there are no guidelines from any societies to guide the perioperative administration of Imeglimin in patients presenting for elective surgery or their continuation/restarting during the post-operative period. Based on the available evidence, it is reasonable to stop it on the day of the surgery/procedure.

## 6. Insulin Icodec

### 6.1. Physiology, Pharmacology, and Mechanism of Action

Under the brand name Awiqli^®^, insulin Icodec is approved for use in both type 1 and T2DM in the EU, Canada, Australia, Japan, and Switzerland [97]. The FDA declined to approve the drug in its May 2024 meeting, pending the submission of more information on the manufacturing process [98].

Insulin itself is not a novel treatment for diabetes mellitus management. However, it has been a struggle to find longer-acting insulin preparations. While NPH insulin (Neutral Protamine Hagedorn) has a half-life of 5–10 h and can be administered one–two times daily, first-generation basal analogs and ultra-long-acting basal insulin analogs have half-lives of 0.5 and 1 day and can be administered once daily. A more frequent need for injections invariably results in poor compliance and subsequent poor diabetes mellitus control in both type 1 and T2DM. Poor compliance in turn results in recurrent diabetes ketoacidosis. The most recent version is once-weekly Icodec, with a half-life of about 1 week, which can increase compliance. It has a fatty acid side chain that is responsible for its prolonged duration of action. Insulin Icodec was made by re-engineering the ultra-long oral basal insulin OI338 [99].

In a multicenter, open-label, treat-to-target phase 2 RCT involving T2DM patients (HbA1c 7.0–10.0%) on basal insulin (total daily dose 10–50 units), three groups were studied. They included Icodec with an initial 100% loading dose (and double first dose), Icodec with no loading dose, and 100 units of insulin glargine once daily for 16 weeks [100]. Switching to once-a-week Icodec resulted in effective glycemic control.

In a meta-analysis that included four RCTs (published from 2020 to 2023), involving 1035 patients with type II diabetes mellitus, Saleem compared insulin Glargine U-100 and insulin Icodec. They recorded similar mean changes in HbA1c (%) and FPG (mg%) [101]. Significantly, there was no significant difference in hypoglycemic episodes.

Mukhopadhyay included seven trials in a meta-analysis comparing once-daily basal insulin analogs to once-weekly basal insulin Icodec. They found a slightly higher risk of overall hypoglycemia and weight gain, without any difference in severe hypoglycemia with once-weekly basal insulin Icodec. Both groups had similar HbA1c control [102].

### 6.2. Perioperative and Anesthesia Implications

The FDA expressed concern with hypoglycemia in its May 2024 committee meeting. Based on the submitted datasets, the FDA safety statistical review concluded that the incidence of level 2 and level 3 hypoglycemia in the phase 3 trials were unacceptably high [103]. As described by the endocrine society, a blood sugar level of less than 70 mg/dL, but more than 54 mg/dL, is labeled level 1 (mild) hypoglycemia. Level 2 (moderate) hypoglycemia is less than 54 mg/dL. Level 3 (severe) hypoglycemia is also less than 54 mg/dL; however, in this case, the person is unable to function because of mental or physical changes due to low blood glucose and requires assistance from another person for recovery due to confusion or unconsciousness [104,105]. Anesthesia providers need to be cognizant of the higher possibility of unexpected and serious hypoglycemia in patients on insulin Icodec. The product’s monograph warns about the risk of hypoglycemia, especially while changing from other insulin products to Awiqli^®^ [106].

There are no specific guidelines available with regard to the perioperative management of care for patients on insulin Icodec.

Table 1 lists all modern hypoglycemia agents and their perioperative implications.

Table 2 and Table 3 discuss the perioperative management of patients on GLP-1 agonists and SGLT-2ia (sodium–glucose cotransporter-2 inhibitors, respectively.

## 7. Conclusions

The availability of multiple drugs with different mechanisms of action is beneficial for patients with diabetes mellitus. Many of these drugs have benefits beyond the control of blood sugar alone. Delayed gastric emptying is the major perioperative concern in patients on GLP 1 analogs, while euglycemia ketoacidosis is the worry in patients on SGLT2 inhibitors. Fortunately, DPP-4 inhibitors and Imeglimin do not have any known risks. The most recent iteration of insulin, Icodec, is very promising. However, it carries slightly higher risk for moderate and severe hypoglycemia. Various societies have published guidelines to address some of the concerns with GLP 1 analogs and SGLT2 inhibitors. While more guidelines are anticipated, it is to be anticipated that other drugs might emerge. Novo Nordisk announced that a phase 3a trial of IcoSema (a once-weekly injection of combined semaglutide (a GLP1 analog) and Icodec) has been completed in patients with T2DM [107]. It is crucial that perioperative care providers are aware of both existing and upcoming developments in the field of diabetes mellitus to provide the best experience for our patients.

## Figures and Tables

**Table 1:** Modern glucose lowering agents and their perioperative implications.

Drug Class/Drugs	Perioperative Implications	Current Recommendation
Glucagon-like peptide 1 analogs (GLP-1A)	Delayed gastric emptying, increased risk of aspiration	AGA, ASMB, ASA, SAGES, and ISPCOP jointly recommend the same decision-making approach—consult procedural, anesthesia, and prescribing care teams, balancing the need for the GLP-1A with individual patient risk. Point-of-care gastric ultrasound, if necessary. If no additional risk factors (e.g., gastroparesis), no need to withhold before the surgery/procedure. Recognize need for modified/extended fasting in some situations.
Sodium–glucose cotransporter-2 (SGLT-2) inhibitors	Sodium–glucose cotransporter-2 (SGLT-2) inhibitors	ACC recommends withholding 3 days before surgery (for canagliflozin, dapagliflozin, and empagliflozin) and 4 days prior to surgery (for ertugliflozin). ADA recommends avoiding in cases of severe illness, in people with ketonemia or ketonuria, and during prolonged fasting and surgical procedures.
DPP-4 inhibitors	Atrial flutter reported in patients with T2DM	DPP-4 inhibitors can be taken as normal both the day preceding and the day of the surgery; patients on variable-rate intravenous insulin infusion should stop DPP-4 inhibitors until eating and drinking normally (working party of the AAGBI). DPP-4 inhibitors should not be administered to hospitalized patients (ADA).
Imeglimin	Appears to be safe	Currently, there are no guidelines. Reasonable to avoid on the day of the procedure
Insulin Icodec	Increased risk of moderate and severe hypoglycemia	Currently, there are no guidelines.

AAGBI—Association of Anaesthetists of Great Britain and Ireland; ADA—American Diabetic Association; AGA—American Gastroenterological Association; ASMB—American Society for Metabolic and Bariatric Surgery; ASA—American Society of Anesthesiologists; SAGES—Society of American Gastrointestinal and Endoscopic Surgeons; ISPCOP—International Society of Perioperative Care of Patients with Obesity; ACC—American college of cardiology.

**Table 2:** Perioperative management of patients on GLP-1 agonists (adapted at Jefferson Health System, Philadelphia, PA, USA).

GLP-1 taken Daily or Week Prior to the Procedure: - WIthhold GLP-1 agonists on the day of the procedure/surgery for patients who take the medication daily. - WithhHold GLP-1 agonists a week prior to the procedure/surgery for patients who take the medication weekly. - Notify Jefferson Perioperative Optimization Center Anesthesiologist for any concerns.			
Day of the procedure considerations: - Consider delaying the procedure if the patient is experiencing GI symptoms such as severe nausea/vomiting/retching, abdominal bloating, or abdominal pain, and discuss the concerns of potential risk of regurgitation and aspiration with the proceduralist or surgeon and the patient. - Continue with the procedure if the patient has no GI symptoms and if the GLP-1 agonist medications have been withheld as advised. - If the patient has no GI symptoms, but the GLP-1 agonist medications were not held, use precautions based on the assumption that the patient has a “full stomach” or consider using ultrasound to evaluate the stomach contents. If the stomach is empty, proceed as usual. If the stomach is full or if the gastric ultrasound is inconclusive or not available, consider delaying the procedure or proceeding using full stomach precautions. Discuss the potential risk of regurgitation and aspiration of gastric contents with the proceduralist or surgeon and the patient. Full stomach precautions also should be used in patients who need urgent or emergency surgery.			
**GLP-1 Agents**			
**Name**	**Brand**	**Half-Life**	**Dosage**
Exenatide	Byetta	2.4 h	Twice-daily injections
Exenatide	Bydureon	7 days	Once-weekly injections
Liraglutide	Victoza/Saxenda	13 h	Once-daily injections
Dulaglutide	Trulicity	90 h	Once-weekly injections
Lixisenatide	Lixumia/Adlyxin	3–4 h	Once-daily injections
Semiglutide	Ozempic	160 h	Once-weekly injection
Oral Semiglutide	Rybelsus	7 days	Once-daily oral administration
Tirzepatide	Mounjaro	120 h	Once-weekly injection
Tirzepatide	Zepbound	5 days	Once-weekly injection
Key Points Patients with severe renal dysfunction should not take GLP-1 agonists. If a GLP-1 agonist is added to a regimen already consisting of Sulfonylurea or long-acting insulin, the patient requires monitoring for hypoglycemia. A decrease in the insulin dose may become necessary, depending on the GLP-1 analog selected. GLP-1 agonists may alter the absorption of warfarin by delaying gastric emptying. An international normalized ratio (INR) should be evaluated and considered while bridging patients off of warfarin. Clinicians should also monitor patients taking GLP-1 agonists if signs and symptoms are consistent with pancreatitis.			

**Table 3:** Perioperative management of patients on SGLT-2i (sodium–glucose cotransporter-2 inhibitors) (adapted at Jefferson Health System, Philadelphia, PA, USA).

Day or Week Prior to Surgery - SGLT2i should be held 3 days prior to surgery and 4 days prior for ertugliflozin. Day of the Procedure - On the morning of surgery, assess the patient for symptoms of nausea, vomiting, and abdominal pain. If positive, obtain a basic metabolic panel to evaluate the anion gap status. - If the patient did not stop their SGLT2is the morning of surgery, obtain a basic metabolic panel. If there is no evidence of metabolic acidosis, then proceed with surgery. Notify the postoperative team to follow the patient for euglycemic metabolic acidosis.
The management strategy is as follows:
SGLT2i Agents and SGLT2i Agents and Other Diabetic Agents
Canagliflozin (Invokana^®^);
Canagliflozin/metformin (Invokamet^®^);
Canagliflozin/metformin XR (Invokamet^®^ XR);
Dapagliflozin (Farxiga^®^);
Dapagliflozin/metformin XR (Xigduo^®^);
Dapagliflozin/metformin XR (Xigduo^®^ XR);
Dapagliflozin/saxaglipitin (Qtern^®^);
Dapagliflozin/saxaglipitin/metformin (Qternmet^®^ XR);
Empagliflozin (Jardiance^®^);
Empagliflozin/metformin (Synjardy^®^);
Empagliflozin/metformin XR (Synjardy^®^ XR);
Empagliflozin/linagliptin (Glyxambi^®^);
Empagliflozin/linagliptin/metformin XR (Trijardy^®^ XR);
Ertugliflozin (Steglatro™);
Ertugliflozin/metformin (Segluromet™);
Ertugliflozin/sitagliptin (Steglujan™).
